# Dynamic STEM-EELS for single-atom and defect measurement during electron beam transformations

**DOI:** 10.1126/sciadv.adn5899

**Published:** 2024-07-17

**Authors:** Kevin M. Roccapriore, Riccardo Torsi, Joshua Robinson, Sergei Kalinin, Maxim Ziatdinov

**Affiliations:** ^1^ Center for Nanophase Materials Sciences, Oak Ridge National Laboratory, Oak Ridge, TN 37831, USA.; ^2^ Department of Materials Science and Engineering, The Pennsylvania State University, University Park, PA 16802, USA.; ^3^ Department of Materials Science and Engineering, Institute for Advanced Materials and Manufacturing, University of Tennessee, Knoxville, TN 37996, USA.; ^4^ Physical Sciences Division, Pacific Northwest National Laboratory, Richland, WA 99352, USA.

## Abstract

This study introduces the integration of dynamic computer vision–enabled imaging with electron energy loss spectroscopy (EELS) in scanning transmission electron microscopy (STEM). This approach involves real-time discovery and analysis of atomic structures as they form, allowing us to observe the evolution of material properties at the atomic level, capturing transient states traditional techniques often miss. Rapid object detection and action system enhances the efficiency and accuracy of STEM-EELS by autonomously identifying and targeting only areas of interest. This machine learning (ML)–based approach differs from classical ML in that it must be executed on the fly, not using static data. We apply this technology to V-doped MoS_2_, uncovering insights into defect formation and evolution under electron beam exposure. This approach opens uncharted avenues for exploring and characterizing materials in dynamic states, offering a pathway to increase our understanding of dynamic phenomena in materials under thermal, chemical, and beam stimuli.

## Teaser

Dynamic states of atomic and defect configurations are autonomously probed at the single-atom level in STEM-EELS.

## INTRODUCTION

Layered materials including graphene, dichalcogenides MX_2_, MXenes, and layered thiophosphates MPS_3_ have attracted considerable attention because of their unique electronic, optical, and mechanical properties (*1*–*5*). Among the multitude of possibilities, an important application of layered dichalcogenides is in quantum emitters, which are essential components of quantum technologies, including quantum communication and cryptography (*6*). Defects in these materials, such as sulfur vacancies, can lead to the creation of localized electronic states that act as single-photon emitters at room temperature (*7*, *8*).

Another promising application of layered dichalcogenides is in catalysis. Transition metal dichalcogenides, such as tungsten diselenide and molybdenum disulfide, have shown potential as electrocatalysts for hydrogen evolution reactions, which are crucial for energy conversion and storage (*9*, *10*). Last, these materials have shown promise in biological sensing applications. For example, they can be used as biosensors for detecting biomolecules, such as DNA and proteins, because of their high sensitivity to changes in the local environment (*11*, *12*). Defects enable sensing capabilities, as they can create localized binding sites for biomolecules and interactions via the surface states affect the light emitting phenomena or overall conductivity, yielding the detection signal (*13*). Even broader spectrum of functionalities emerges in multilayer systems, where orchestrating the interactions between dissimilar layers via twisting and controlling defect populations opens a virtually intractable design space for quantum materials, catalysts, and biological systems.

However, the critical element for understanding these functionalities is the knowledge of structure-property relationships on a single-defect level. After two decades since the broad introduction of aberration-corrected scanning transmission electron microscopy (STEM) (*14*, *15*), probing local structure via STEM and extracting local chemical information via core-loss electron energy loss spectroscopy (EELS) and quasiparticle excitations via low-loss EELS have become almost routine. However, until now, these methods could be used only for a limited number of scenarios, where the material structure remains relatively stable during hyperspectral data acquisition and the defects of interest are sufficiently abundant. This limitation stems from the fundamental operation principle of STEM, based on manual identification of objects of interest by a human operator, configuring the EELS experiment over the object of interest, and subsequent manual data analytics. Consequently, the throughput of these experiments is very low, and the effective dose over the region of interest (ROI) is very high.

The second limitation of this approach is that only stable, already-present defects in the material can be explored. Taken jointly, these factors lead to the fact that very few defects in two-dimensional (2D) and layered materials are studied in depth via STEM-EELS, and often hours of work of a qualified operator lead to reliable measurements for only a few defect types. Crucially, the measurement of the intended ROI in many samples turns out to represent an entirely different structure than was initially observed due to the electron beam altering the structure and chemistry during the acquisition. This effect is compounded by the recent and perhaps renewed interest in off-axis EELS geometry (*16*–*18*), where acquisition times must be markedly increased—from milliseconds to seconds—meaning that substantial electron dose must be imparted to the specimen to collect enough scattered electrons. This collection geometry has been shown to be successful in a relatively few number of cases where the sample is extremely stable under the beam, for instance, vibrational structure of thin-film superlattice interfaces (*19*), and detecting single-atom vibrational spectroscopy (*16*, *20*, *21*) and more recently even in distinguishing different chemical bonding signals between different silicon dopants in graphene (*18*). However, in the latter, substitutional defects in graphene are widely known to move under the beam (*22*–*24*).

We note that the exploration of the structure-property relationships in STEM-EELS can also be performed dynamically. In many layered materials, beam damage effects can lead to slow changes in chemical bonding network, forming point and extended defects, nucleating secondary phases, and eventually leading to material degradation. These processes are often slow, and multiple hundreds of STEM images containing defect configurations can be obtained. These systems are unsuitable for the classical grid-based EELS measurements, because these effective doses are comparable to those during imaging and hence the system is not stationary.

Here, we have developed the rapid object detection and action system (RODAS) workflow combining the STEM imaging at low-dose conditions and deep learning to identify and classify defects on the fly, followed by EELS measurements in the designated locations, thus enabling the construction of dynamic library of defect types during the STEM experiment.

## RESULTS

### Rapid object detection and action system

Acquisition of EEL spectra at various defect and dopant sites in 2D materials is often a challenge. This is because grid-based spectrum imaging imparts too much electron dose, and therefore, the desired structure to measure is altered or destroyed. Correspondingly, an intelligent detection and beam control scheme is required to capture the signals. It has been proposed that deep convolutional networks can be used to identify objects of interest in the streaming data (*25*, *26*). However, for classical deep convolutional neural networks (DCNNs), both the object detection and beam control have been elusive, and feature detection was too sensitive to hyperparameters for it to be useful on the fly. Recently, however, ensemble learning allowed to markedly improve the robustness of model predictions (*27*), making real-time analysis possible (*28*, *29*). Within the past few years, controlling the electron beam position (and other microscope parameters) has been made possible by manufacturers such as Nion and JEOL by providing Python-based Application Programming Interfaces (APIs) to end users (*30*, *31*). Aside from this, custom control schemes also exist that may be implemented to virtually any instrument where an API is not accessible.

The classical STEM-EELS experiments typically begin with an overview structural image, usually a high-angle annular dark field (HAADF)–STEM image. From this relatively fast scan, an ROI is selected, and a spectrum image acquisition begins, in which a spectrum is collected at a grid of points defined by the ROI, where the pixel size is typically larger than its HAADF counterpart—a result of the increased needed dwell time. In this manner, the HAADF signal can be synchronously collected, meaning that with almost absolute certainty, it is clear what structure has been measured. Aside from decreasing the dwell times as much as possible, a standard method for reducing the total dose and therefore the effect of the beam is to reduce the number of total pixels acquired, i.e., increase the physical size of each pixel. This may be successful if the feature sizes are comparable to the pixel size; however, when probing atomic structures, this option loses its feasibility. One final approach might be to use pixel sizes that are appropriate for realizing structure, but instead of acquiring a single-spectrum image with pixel time *t*, instead acquire *n* spectrum images with pixel time *t/n*. Several groups have used this method successfully but to compensate for specimen spatial drift (*17*, *32*), which might be particularly useful at cryogenic temperatures. Ultimately, this and other approaches still impart far too much electron dose to the specimen that prevents an accurate extraction of the spectral signature of specific defect sites that are generally not extremely robust to the beam.

Alternatively, instead of an entire spectrum image, one may acquire a point spectrum at a chosen location in the overview HAADF-STEM image, by manually positioning the electron beam and recording the spectrum. While potentially dose efficient, this approach suffers multiple downsides: First, it is not necessarily repeatable or reliable to position the electron beam by hand. Scan distortions (*33*) and nonrepeatable human movements can result in major alignment problems. Second, a simultaneous structural image is not obtained, signifying that it is unknown whether the specimen has been affected by the beam or drifted from the intended probe location.

**Fig. 1.**
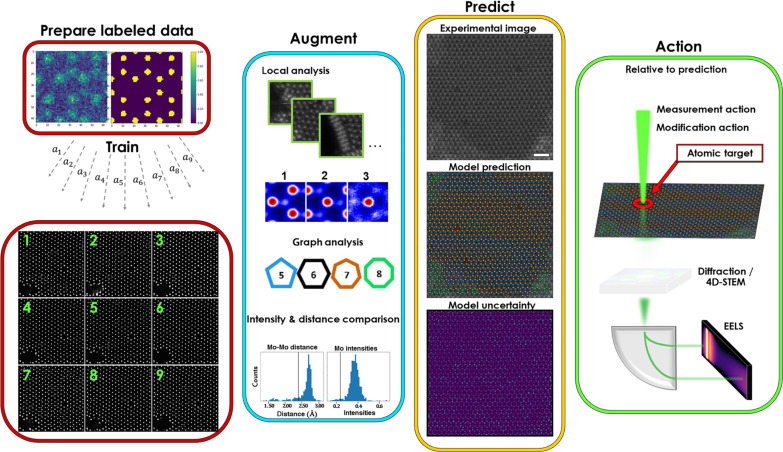
Ensemble networks to predict atomic coordinates and classes observed in experiment in real time. Initial set of labeled training data is trained with multiple different random initializations, *a_i_*, where ensemble prediction is average of each model prediction within ensemble. To generalize to experimental conditions and handle multiple classes occurring because of structural changes, ensembles are augmented (retrained) with additional classes encountered during experiment by labeling experimental data (with some manual adjustments), then using it as new training data.

We address these challenges by proposing the workflow that identifies atomic configurations dynamically as they form under the action of the electron beam and collects EELS data only on objects of interest. This approach is enabled by a robust feature identification method—a trained ensemble of DCNNs in what is known as ensemble learning iterative training (*27*). The ensemble networks allow on-the-fly atomic identification and classification and is the core-enabling aspect of this work, and their application here is depicted in Fig. 1. The ensemble models are typically trained offline using simulated training data and then augmented with fresh experimental images that may contain additional classes that are difficult or intractable to simulate. We do not, however propose to have a trained model that covers every possible defect class (size of combinatorial defect space is vast); rather, metrics like interatomic distances or atomic intensities can be used to classify defects on the fly and to avoid having to retrain a new model for each additional emerging class. Augmentation here means using the first ensemble model to predict atomic classes of fresh experimental data and using additional analyses (local structure analysis, intensity comparisons, graph analysis, etc.) in a partially automated approach to refine and classify any new classes that were not contained in the previous model. These newly classified experimental data serve as the training set for a new ensemble model. Note that the augmentation step may be performed during the experiment but can require a considerable amount of training time; in this example, augmentation and retraining took approximately 30 min using a single mid-tier graphics processing unit (GPU), but this can vary on the basis of number of features in an image and number of pixels per feature, where here, an atom is a feature.

**Fig. 2.**
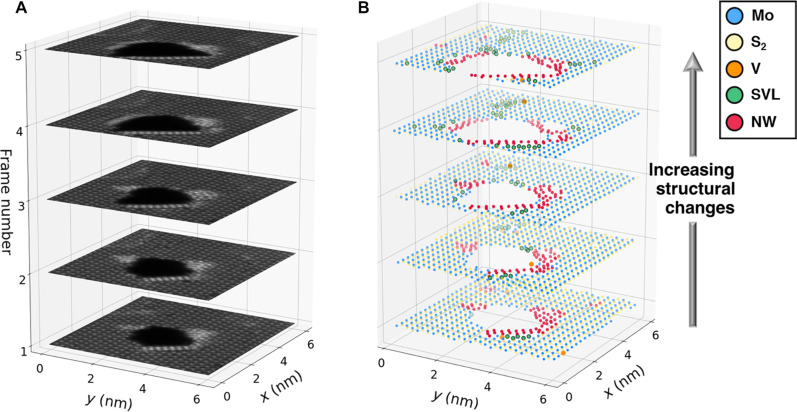
Classification of system dynamics under the electron beam. Time series HAADF-STEM images shown in (**A**) where the atomic coordinate classification is shown in (**B**). With each additional frame, more electron dose causes additional structural transformation. Extant and emerging classes are classified on the fly using metrics such as neighbor distances, intensity comparisons, or other local analyses.

It is highlighted that machine learning (ML) applied during active and live experiments is different from the classical ML used on static data. This is due to the need to execute ML inference fast and with minimal input from the operator. We further note that classical benchmarking is inapplicable in this case because of the active nature of the process, and rather, the human operator steers the ensemble performance. For full ensemble prediction to be completed in a reasonable time frame (e.g., within hundreds of milliseconds), one needs to consider appropriate GPU resources for prediction with each model within the ensemble: Current off-the-shelf graphics cards can be used for this purpose. Otherwise, a single model from the ensemble can be chosen by the operator, which relaxes the need for expensive GPU computations, at the risk of being slightly less robust. Here, the HAADF-STEM image serves as an input to the ensemble network, which provides atomic coordinates (semantic segmentation) and classes as an output. Considering now that there can exist beam-induced transformations, we refer to Fig. 2, where system dynamics of MoS_2_ are observed under the beam in sequential time steps. The ensemble is used to identify and classify atoms and, based on heuristics such as local bonding environment, intensities, and distance metrics, can be further classified into defect classes. Combined with flexible probe positioning and access to the EELS camera acquisition, this also allows the spectra from detected atomic sites to be acquired in a fully automatic fashion. Note that not all atomic sites will be measured, but generally only the defect sites and usually a small number of regular (nondefect) sites that serve as a basis for comparison.

### Dynamics assessment

The primary limitations of the proposed approach stem from the potential probe or specimen drift, the nonideality of scanning systems, and uncertainty on whether the structure has changed during a measurement or not. Even if the HAADF intensity is simultaneously acquired with EELS spectrum, it represents the information from a single pixel only, and it is not possible to determine local structure from a single pixel. To alleviate this problem, here, we acquire a second fast, and minimal dose HAADF-STEM image after the spectra are collected, which can then be compared with the original image to determine whether a beam-induced change or drift had occurred. Cross-correlation of images is especially difficult or unreliable if there is a beam-induced effect causing substantial contrast change; therefore, in this case, we used the motion of all atomic coordinates—or a subset of coordinates, such as V substitutions, that can serve as good fiducial markers because of their stability (*34*)—between frames to detect image shift and change. Contamination in the field of view can also serve as a suitable marker for ascertaining drift. Alternatively, one may create image patches at each target coordinate in the current and subsequent frame and compare the image patches in time; this assessment may be done by cross-correlation. Note that this step can safely be performed after experiment, implying that the comparison method can be modified and finely tuned. At this stage, a pair of images will have been obtained, with a small number (~10) of various spectra collected from specific atomic sites relative to the first image. More data, and therefore statistics, can be collected immediately by simply repeating this process, using the second image to predict atomic coordinates and classes with the ensemble network, acquisition of more EEL spectra at specific atomic sites, followed by a third HAADF-STEM image, and so on. Critically, in materials that undergo beam-induced transformations such as MoS_2_, which is used in this study, new atomic and defect classes may arise during these processes and are classified in addition to the other standard atomic classes such that they can also be probed as they appear. This is repeated as much as possible, where the process (i.e., number of points visited, entire duration, etc.) is dictated by the beam sensitivity of the specimen as well as the sample drift. In other words, the total time for a set of spectrum acquisitions during one step should be sufficiently less than the current drift rate. Similarly, each step should also attempt to use as little as dose as possible, meaning that the number of measurement points should be small and that simultaneously points should be separated in space to avoid overlapping dose; the latter is usually avoided by using atomic sites as measurement coordinates.

It should be mentioned that the instrumental control via Python commands is limited by the operating system’s current demands, and process timing cannot be guaranteed; in other words, the dwell time of the beam is achieved by using a Python command, which can be set equal to the camera exposure time. One might assume that iterating over coordinates in this fashion should perform well; however, because of unpredictable operating system processes, additional delay on the order of hundreds of milliseconds can be accumulated with each new positioning command. While the EEL spectrum will correctly represent the appropriate camera dwell time, the beam may ultimately end up dwelling much longer than expected. In the case presented here, dwell times are already >1 s, therefore the extra possible hundreds of milliseconds are not a substantial change in terms of dose. If one decides to use this approach with much shorter beam dwell times, then hardware synchronization must be used.

**Fig. 3.**
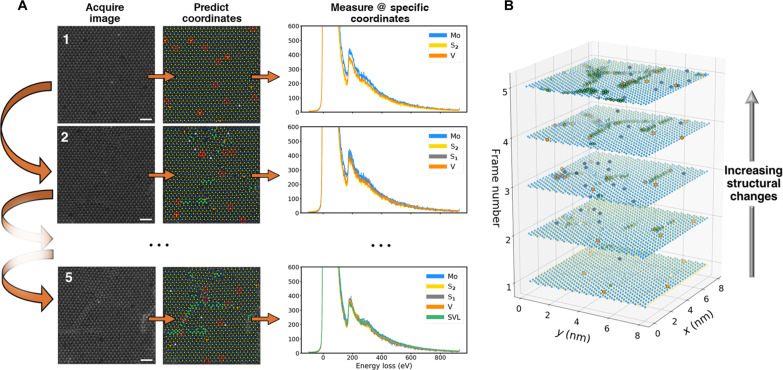
Automated site-specific atomic EELS experiment during beam transformations. During a microscope session (**A**), a HAADF-STEM image is acquired, atomic coordinates are predicted, and several EEL spectra are collected (shown in red circles) from select different classes. This is repeated several times to induce structural changes as well as gather more statistics or until the microscopist forces it to end. Post acquisition analytics (**B**), atomic coordinates from each frame are used as a metric for both determining whether subsequent frames are aligned satisfactorily as well as whether the specimen has degraded, transformed, or suffered from contamination. If unsatisfactorily aligned, those spectra are discarded, and the remaining classes are averaged.

Last, the sequence of image pairs and measurements are analyzed for drift and changes to the sample after the experiment. A crucial concern is to confirm that we measure what we expected to measure; in other words, during a measurement, did the specimen transform, damage, or drift too far? If so, we do not wish to include that measurement, as it represents a combination of two or more structural phases or simply something that is uncertain. It will more than likely be obvious during the experiment when this occurs, but this is generally not a concern because this approach is fully automated anyway. If in postprocessing it is found that a change to the specimen has occurred during the sequence or too large a drift was present, then these measurements are discarded, as they are not representative of the intended structure. This evaluation is done by comparing the second image to the first image: After image registration, if a hole forms or change occurs in image #2 where we measured an atom or defect in image #1, then we must discard this measurement. The overall workflow is represented in Fig. 3, where it is emphasized that the beam induces sulfur vacancies (which are themselves a defect class, denoted by S_1_) during the imaging and measurement processes that eventually form ordered sulfur vacancy lines (SVLs). With the emergence of new structural classes, new spectral signatures may arise; therefore, these new classes are dynamically measured. Structural changes other than SVLs are possible, notably MoS edge reconstructions (see Fig. 2), and are measured and discussed later.

### Point spectrum EELS and off-axis EELS of single atoms and defects

While the proposed framework is universal, here, we focus on the applications to STEM-EELS. Several regimes of EELS exist, which exhibit differences in dwell times and electron optic configurations. We consider the following two geometries: on-axis and off-axis EELS. On-axis EELS is the standard geometric configuration where the scattered bright-field (BF) disc enters the EEL spectrometer aperture. Comparatively, in off-axis EELS, the scattered dark-field disc instead enters the spectrometer. The motivation for this is similar logic as to why the dark-field signal is used for structural imaging—electrons can undergo primarily either impact scattering or dipole scattering, where the smaller the scattering angle, dipole scattering is more strongly favored, and therefore the signal is more delocalized.

In the on-axis EELS configuration (small scattering angles), dipole scattering dominates, which incidentally enables the so-called “aloof” EELS, where the beam can be placed tens or even hundreds of nanometers—depending on energy loss—from a specimen and the electrons still may lose energy via the dipole scattering process (*35*). While the on-axis EELS signal is several orders of magnitude larger than that of off-axis EELS, generally the trade-off lies in the localization of the signals. In general, at core loss energies (>50 eV), the on-axis EEL signal is strongly localized to atoms, so off-axis is not necessarily required for core loss energies. At lower energy losses, and particularly in the regime of optical and vibrational excitations, however, the on-axis signals can become highly delocalized (*36*). Consequently, to study the relatively low-energy vibrational, optical, and electronic excitations of single atoms or defects, it is increasingly necessary (in general) to use an off-axis collection geometry.

**Fig. 4.**
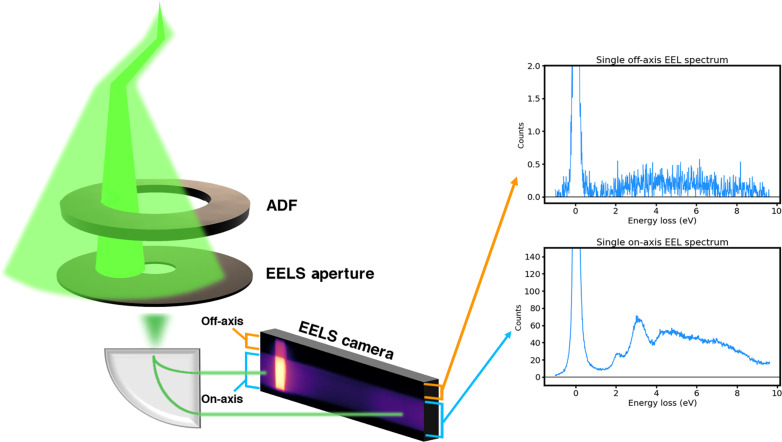
On-axis and off-axis: Monochromated STEM-EELS. Both on- and off-axis signals enter the spectrometer and strike the detector and are easily distinguished in the camera view, therefore two separate vertical regions can be integrated to split the on- and off-axis signals. Background subtraction is not performed, as it is difficult to model the zero loss peak characteristics for a monochromated source. Raw spectra for one atomic class (Mo) are shown for both the on- and off-axis EELS scenarios. Note the difference in counts between the two configurations.

The off-axis configuration can be achieved by electrically displacing the aperture with projector lenses, or by using an annular spectrometer aperture. Here, we use the former, as it allows simultaneous collection of BF and dark-field information by partially displacing the aperture, such that one portion of the camera receives the BF information while the remaining portion of the camera receives the dark-field information. In this way, the full 2D EELS camera is collected for each measurement and is separated into an on-axis and off-axis portion in postacquisition analysis, as shown in Fig. 4. In principle, separation can be done during the experiment but was not required here.

A point of contention when using the electrically displaced EELS spectrometer aperture is that it results in a known asymmetric annular dark field (aADF) intensity distribution. A portion of the BF disc may now be partially striking the HAADF detector, which is normally restricted to scattering angles on the order of 80 to 200 mrad. Because a markedly larger number of electrons is contained in the BF signal relative to higher scattering angle signals, this aADF signal is primarily dominated by the portion of the BF disc that impinges upon the detector. Consequently, the observed structural image more closely resembles annular BF or medium angle annular dark field signals, but this largely depends on the amount of shift to enter this operating geometry. Practically, this has the effect of changing the image contrast. In other words, this type of aADF signal is considered “out of distribution,” i.e., the training dataset for most ensemble networks up until this point generally has assumed that the image contrast is close to that of what the HAADF signal produces. This simply means that the training data must more closely match experimental conditions, as is the usual case for model training. Last, to access the low-energy excitation regime in STEM-EELS, a form of monochromation is needed. While energy resolutions down to 2.6 meV (at 20-keV primary energy) have been reported (*37*), the monochromator itself imparts several additional aberrations to the electron probe. This also can affect image generation, making repeatable atomic resolution challenging. In this work, an energy resolution of around 145 meV [see fig. S1 for zero loss peak (ZLP)] was used to observe excitations down to about 1 eV as the bandgap in MoS_2_ is near 1.6 eV; this also allows a larger effective beam current compared to a more strongly monochromated probe. For core loss experiments, an energy resolution of about 1.5 eV was used so that we could simultaneously observe a large energy loss region (0 to ~850 eV).

A Nion monochromated aberration-corrected STEM, or MACSTEM, operated at 60 kV equipped with a Dectris ELA detector was used in these automated EELS experiments. Even with electron-counting, direct electron detector (Dectris ELA), the number of electrons collected from a monochromated dark-field signal is extremely small, meaning that pixel dwell times on the order of seconds are required to capture a reasonable amount of signal. This is approaching the fundamental limit of collecting every appropriately scattered electron while trying to balance and minimize any beam-induced effect; hence, an intelligent positioning scheme is needed to reduce any unnecessary exposure.

## DISCUSSION

As a model system, single-layer 1% V-doped metal-organic chemical vapor deposition (MOCVD)–grown MoS_2_ is explored (*38*). It is well known that MoS_2_ is sensitive to the electron beam in the STEM even at accelerating voltages down to 30 kV; this is due to multiple damage mechanisms by the electron beam. Because MoS_2_ is a semiconductor, both knock-on and radiolysis contribute to the overall damage; however, it does remain stable for a handful of HAADF-STEM images to be taken, corresponding to doses approximately on the order of 10^6^ − 10^7^ *e*^−^/Å^2^ before sulfurs are ejected and various defect structures begin to form (*39*). Encapsulation of MoS_2_ with graphene has been shown to protect against damage; however, this involves multiple additional steps and introduces more complexity in sample preparation as well as possible different effects on electronic structure.

Upon finding a suitable ROI, the automated experiment begins by acquiring an overview HAADF, here shown with a field of view of 6 or 8 nm, followed by atomic coordinate prediction and classification, and last, a series of site-specific EEL spectra are collected at several of each class of atom. In this case, we have up to five atomic classes: Mo, disulfur, V-substitutional defect, a SVL defect, or an MoS edge reconstruction—here referred to as nanowire (NW). Aside from the vanadium substitutional defect, all other defects here are in some way caused by S vacancies, where coherent ordering of these can cause additional defect classes, like SVLs and NWs, to emerge. The algorithm was chosen such that a total acquisition time does not exceed 20 s (for drift considerations). In addition, it seeks to acquire multiple spectra from each class that is detected. Here, we collect the full 2D EELS camera, and segment it into on- and off-axis contributions after acquisition. As mentioned previously, the spectral data are discarded if either too large of a specified drift has occurred between frames, or structural changes have occurred; in other words, the spectral data are no longer representative of the perceived structures (they dynamically change through the experiment).

**Fig. 5.**
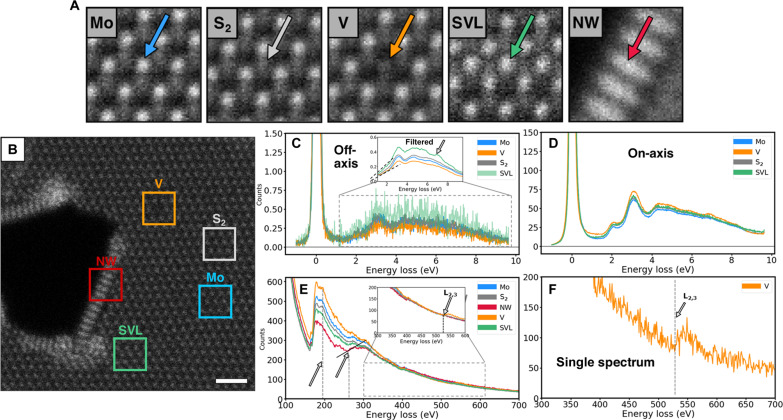
Single-atom and defect EELS. Collection and averaging of multiple experiments after postexperiment verification. Atomic and defect classes are probed as they appear throughout experiment, where the beam induces structural transformations. Class-averaged image patches (1-nm field of view) for each class are shown in (**A**), with arrows indicating from where the spectra were nominally collected, where representative HAADF-STEM containing all classes simultaneously is shown in (**B**). Both off- and on-axis low-loss signals are collected simultaneously (**C** and **D**, respectively) and separated by integrating different regions of the detector as shown in Fig. 4. Inset of (C) shows Savitzky-Golay filtered spectra of the boxed region, with dashed lines highlighting differences in slope near band edge ~1.6 eV. Core-loss spectral region presented in (**E** and **F**), where multiple spectral differences between the individual atomic and defect classes are observable; see arrows for highlighted differences. Inset in (E) shows zoomed in region of boxed region highlighting the L_2,3_ edge of the averaged vanadium signal. Single spectrum from a single vanadium substitutional defect shown in (F), where L_2,3_ edge is clearly visible, whose acquisition time was 1 s. Scale bar, 1 nm (B).

After repeatedly performing these automated experiments, the EEL spectra are aligned and averaged separately for each class. These results are presented in Fig. 5, where the following conclusions can be made. First, the low-loss EELS in the on-axis configuration (Fig. 5D) has almost indiscernible differences aside from a slight increase in intensity across the entire spectrum, but this can be explained from the effective lower Z of vanadium. This motivates the use of the off-axis EELS configuration, especially for defect structures whose local properties are oftentimes elusive because of the masking influence of highly delocalized dipole scattering. Therefore, to reduce the effect of dipole scattering, the off-axis configuration is used. The off-axis regime (Fig. 5C), after appropriate filtering (Savitzky-Golay), does reveal a substantial difference in the SVL low-loss spectrum: The increased intensity in the 2- to 10-eV range may indicate a higher local density of available states—in other words, increased metallic behavior. However, this could also be a Z-contrast effect, as there is an increased (off-axis) ZLP intensity for the SVL relative to other classes (see fig. S4). Fortunately, there is a difference in the onset slope of the band edge near 1.6 eV; this is highlighted in the inset of Fig. 5C and indicates differences in bandgap energies which may be extracted by linear or other fitting methods (*40*). There is also additional peak structure in the SVL spectrum near 7 eV that may also indicate the presence of a defect-related electronic transition, but it is difficult to be quantitative without direct comparison to theoretical calculations. In any case, this modification of the local electronic structure implies a more metallic behavior, which is consistent with density functional theory calculations on SVL structures (*41*).

Second, there are also differences in the core loss regime among the different classes. Here, the NW edge reconstruction is highlighted; these have also been predicted to exhibit metallic behavior (*42*, *43*), and their formation through electron beam irradiation and in situ thermal annealing has been extensively investigated (*44*, *45*). The EELS structure near 260 eV is most apparent, where the edge is stronger (positive slope, seen in Fig. 5E) relative to the others, implying a larger presence of unoccupied d-states, which makes physical sense based on the reduced sulfur coordination and different stoichiometry of the MoS NW. Moreover, observation of the vanadium substitutional defects shows that the vanadium L_2,3_ edge is clearly visible here; even for a single point spectrum from a single vanadium atom (Fig. 5F), the edge is clear. Note that the reduced counts themselves in the energy range from 170 to 300 eV may be misleading as different amounts of elastic scattering may contribute to this (Z contrast effects), therefore no claim is made based on those particular differences. For insight into the spectral statistics and error metrics, figs. S3 and S4 show the standard deviation of all spectra contained within each class for the low-loss and core loss cases, respectively. This is a nontrivial comparison however due to the fact that many of the intensity differences among spectra of the same class can be attributed to the ZLP (also shown). This suggests that intensity differences by themselves do not necessarily indicate changes to electronic structure, but changes in slope and appearance of additional peaks and edges do. Intensity differences within the same class are primarily expected to arise because of slightly different positions of the electron beam relative to the atom core. Subsets of individual core loss spectra are also shown in fig. S5 where further evidence is presented that slopes and loss edges within a given class are consistent and repeatably observed.

The acquisition times per pixel here are on the order of hundreds of milliseconds up to 1 s, where a nominal probe current of about 30 pA was used. These parameters were chosen to obtain enough energy loss counts. Performing traditional EELS mapping (spectrum imaging) with enough dwell time and beam current to detect these features would be impractical and almost certainly impart too much dose to properly detect the signatures of these defect structures. If the dose is reduced such that the material is not disturbed or destroyed by the beam during acquisition of a spectrum image, then not enough detector counts—even with a direct electron detector—are present to make physical conclusions. Figure S2 shows such an experiment where the detector only sees an average of less than 1 detector count at the L_2,3_ edge energies for a pixel corresponding to the V substitutional defect. This illustrates that attempting to manually probe such dynamic processes, whether by spectrum imaging or manual point spectroscopy, is highly uncontrollable, dose intensive, and not reproducible. RODAS therefore provides a systematic and controlled approach for extracting the behavior of these sensitive dynamics.

These experiments can be similarly performed in practically any EELS regime under the realization that the experimental conditions responsible for image generation should closely match the image training data for feature detection. Future experiments are required at lower energy (stronger monochromation) to observe any vibrational differences at these scales and how they might be useful for phononic materials and metamaterials mediated by defect structures. An alternative method could be that instead of acquiring individual point spectra at the detected locations, to acquire a small spectrum image at each target location. Perhaps in this way, a window large enough to capture any structural changes could be used; however, additional electrons will be detrimentally deposited into the neighboring structures.

To summarize, here, we propose and realize an approach for the exploration of the structure-property relationships of complex systems based on the dynamic STEM-EELS measurements of the materials undergoing beam-induced transformations. Classical approaches aim to minimize the beam damage via lowering beam energy or doses. Here, we identify the defects as they form and collect only the data from the defects or atomic classes of interest. Practically, this approach can be trivially extended to prioritize exploration of certain defect classes, focus on emergence of new defects, or balance sampling across possible structural units.

It is again noted that these experiments are not limited to STEM-EELS and are easily adapted to 4D-STEM (i.e., differential phase contrast, nanobeam electron diffraction, etc.), cathodoluminescence, energy dispersive x-ray analysis, etc., and is especially useful in experiments where dwell times are long enough to cause undesirable beam-induced changes. While the primary focus of this work is to enable studying sensitive defects and structures at the atomic scale, in principle, it is not limited to these sometimes difficult-to-reach scales, or even the STEM, but with the appropriate training and accessibility to system controls, is applicable to a wide variety of microscope modalities.

## MATERIALS AND METHODS

### MoS_2_ synthesis

V-doped MoS_2_ films were synthesized on c-plane sapphire substrates using a custom-built MOCVD system. Mo(CO)_6_ (99.99% purity, Sigma-Aldrich) and V(C_5_H_5_)_2_ (99.99% purity, Sigma-Aldrich) powders are placed into stainless steel bubblers and serve as the Mo and V precursors, respectively. During synthesis, the pressure inside the bubblers are kept constant at 735 torr, and the temperature is set to 24°C for Mo(CO)_6_ and 50°C for V(C_5_H_5_)_2_ to maintain a constant precursor vapor pressure. Concentration of Mo and V precursor in the growth chamber can be tightly controlled by introducing precise flow of H_2_ carrier gas through the bubblers. A high-purity H_2_S gas lecture bottle (99.5%, Sigma-Aldrich) is used as the sulfur source. Growths are carried out at a growth temperature of 1000°C and a pressure of 50 torr, using a multistep growth process as previously described (*38*).

To transfer the films onto transmission electron microscope (TEM) grids, the samples were first coated with a layer of poly(methyl methacrylate) (PMMA) (Micro Chem. 950 K A6) using a two-step spin casting process (step 1: 500 rpm for 15 s; step 2: 4500 rpm for 45 s). Next, the PMMA/V-MoS_2_ stack is delaminated from the as-grown sapphire substrate by immersing it in a 30% (w/v) KOH solution heated to 90°C. The free-standing film is then fished out of the KOH bath and rinsed through a series of deionized water baths. Using a Buchner funnel apparatus filled with water, the film is gently allowed to drape onto the TEM grid, aided by gravity. PMMA is dissolved by exposing the TEM grids to acetone vapor for 4 hours.

### Electron microscopy

A Nion UltraSTEM100 was used in these experiments, using a 60-kV electron beam with nominal probe current of 30 pA and semiconvergence angle of 30 mrad. For low-loss EELS experiments, a Nion monochromator was used to moderately monochromate the source providing 145-meV full width at half maximum of the ZLP. For off-axis conditions, the postspecimen beam was electrically shifted by about 20 mrad so that one-third of the on-axis signal entered the spectrometer entrance aperture. A Nion IRIS spectrometer was used in conjunction with Direct Electron’s ELA detector. Customization of beam positioning protocols was done using the available API within Nion Swift, the user interface associated with the Nion instruments.

## Supplementary Materials

### This PDF file includes:

Figs. S1 to S5 Data Repository
