# Supplementary material for: Dynamic STEM-EELS for single-atom and defect measurement during electron beam transformations
Source: Sci Adv. 2024 Jul 17;10(29):eadn5899. doi: 10.1126/sciadv.adn5899 (PMC466940; doi:10.1126/sciadv.adn5899)
Supplement: Supplementary file 1 — Figs. S1 to S5 Data Repository [file sciadv.adn5899_sm.pdf]

Science Advances
AAAS

Supplementary Materials for

Dynamic STEM-EELS for single-atom and defect measurement during electron beam transformations

Kevin M. Roccapriore *et al.*

Corresponding author: Kevin M. Roccapriore, roccapriorkm@ornl.gov;
Maxim Ziatdinov, ziatdinovmax@gmail.com

Sci. Adv. **10**, eadn5899 (2024)
DOI: 10.1126/sciadv.adn5899

This PDF file includes:

Figs. S1 to S5
Data Repository

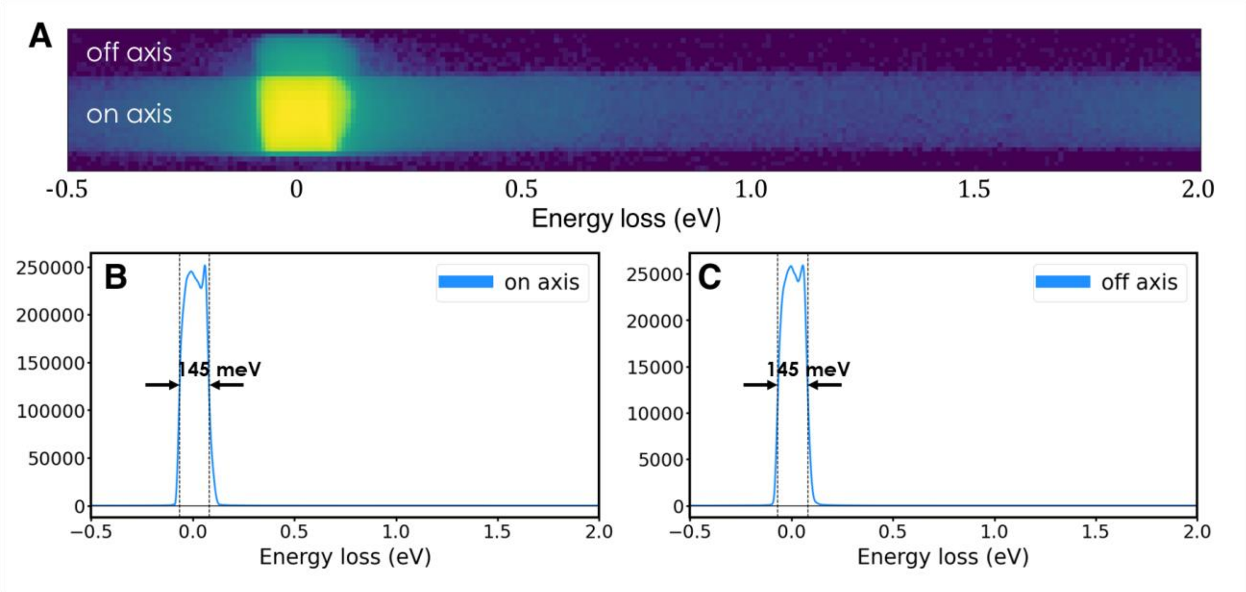

Figure S1. Typical (partially) monochromated low loss 2D EELS camera image (**A**) which is integrated in the vertical direction into an on-axis (**B**) and an off-axis EELS spectrum (**C**). The FWHM of each ZLP is shown, but the tails of the ZLP are drastically reduced due to the monochromation.

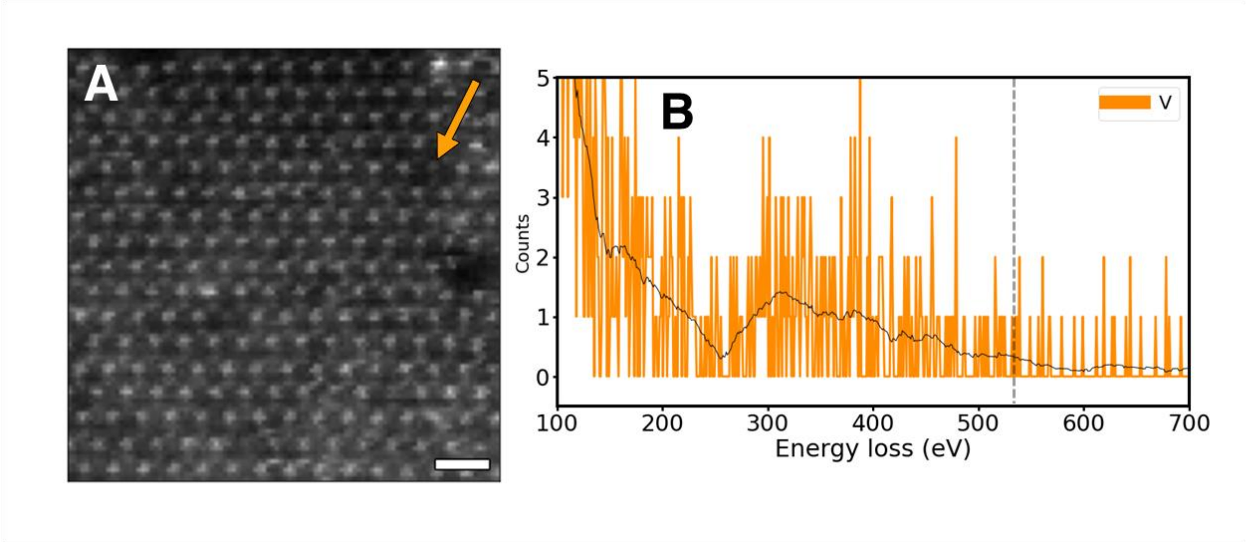

Figure S2. Traditional EELS spectrum imaging (SI) is performed in a region of MoS_2 containing vanadium substitutional defects. Per pixel dwell time is kept short enough so that no beam induced changes occur. The simultaneously acquired HAADF image is shown in (**A**), where the selected EEL spectrum (orange arrow in (**A**)) is presented in (**B**). The $L_{2,3}$ edge is marked with a vertical dashed line for convenience. The raw signal is filtered using a Savitzky-Golay filter to aid in analysis, but the number of counts is too low in this energy region - the average counts are less than 1 detector count per pixel. Note that a direct electron detector is used. Scale bar in (**A**) 5 angstroms.

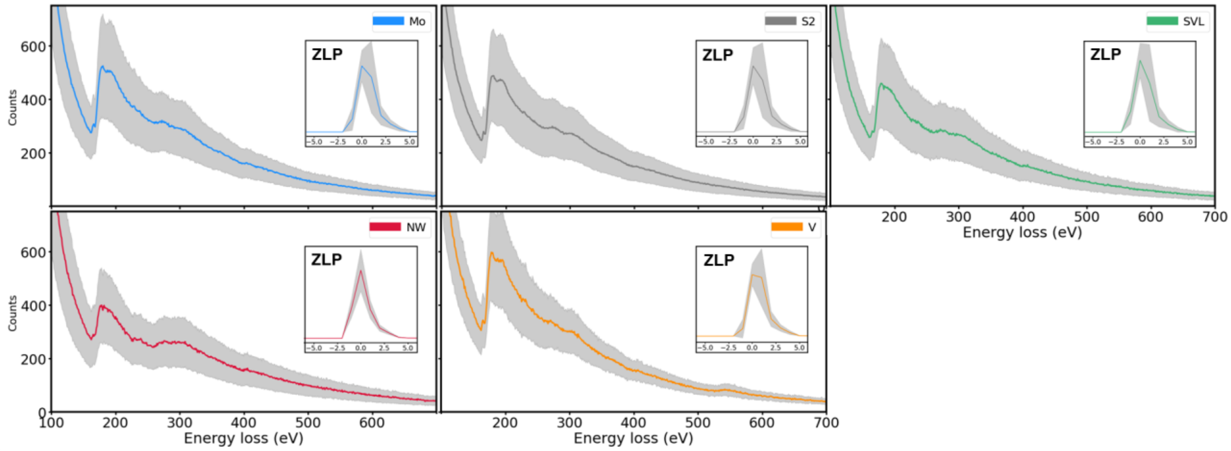

Figure S3. Distribution of classified on-axis core-loss spectra. Average spectrum for each class is shown (identical curve as in Figure 4 in main text), with the shaded region representing the standard deviation. Insets show the ZLP also with shaded standard deviation, showing that a large portion of the intensity deviation in the core loss is related to the ZLP and does not necessarily electronic feature differences. Changes in slopes, however, do indicate physical differences and are not resulting from ZLP differences – i.e., positive slope around 260 eV for the NW class.

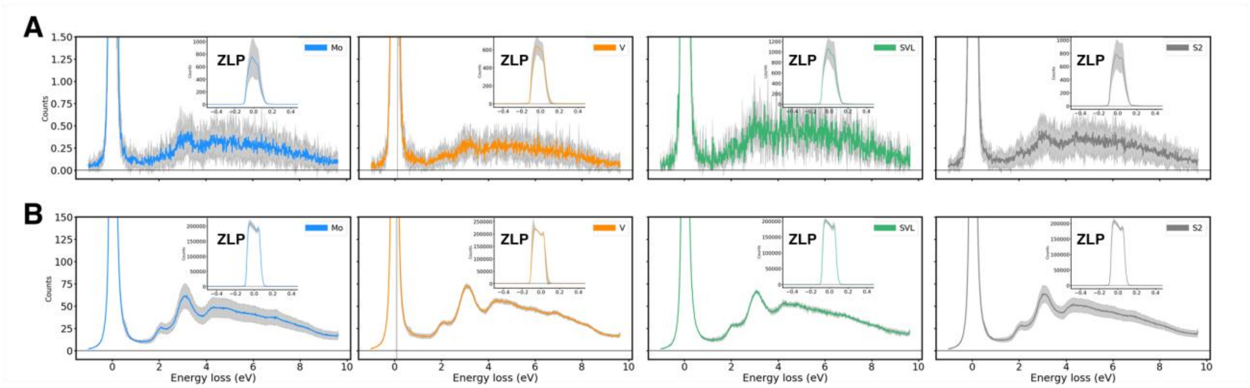

Figure S4. Distribution of classified low loss spectra. Off-axis signals shown in (**A**), while on-axis shown in (**B**). Average spectrum for each class is shown (identical curve as in Figure 4 in main text), with the shaded region representing the standard deviation. Insets show the ZLP also with shaded standard deviation, showing that a large portion of the intensity deviation in the core loss is related to the ZLP and does not necessarily electronic feature differences.

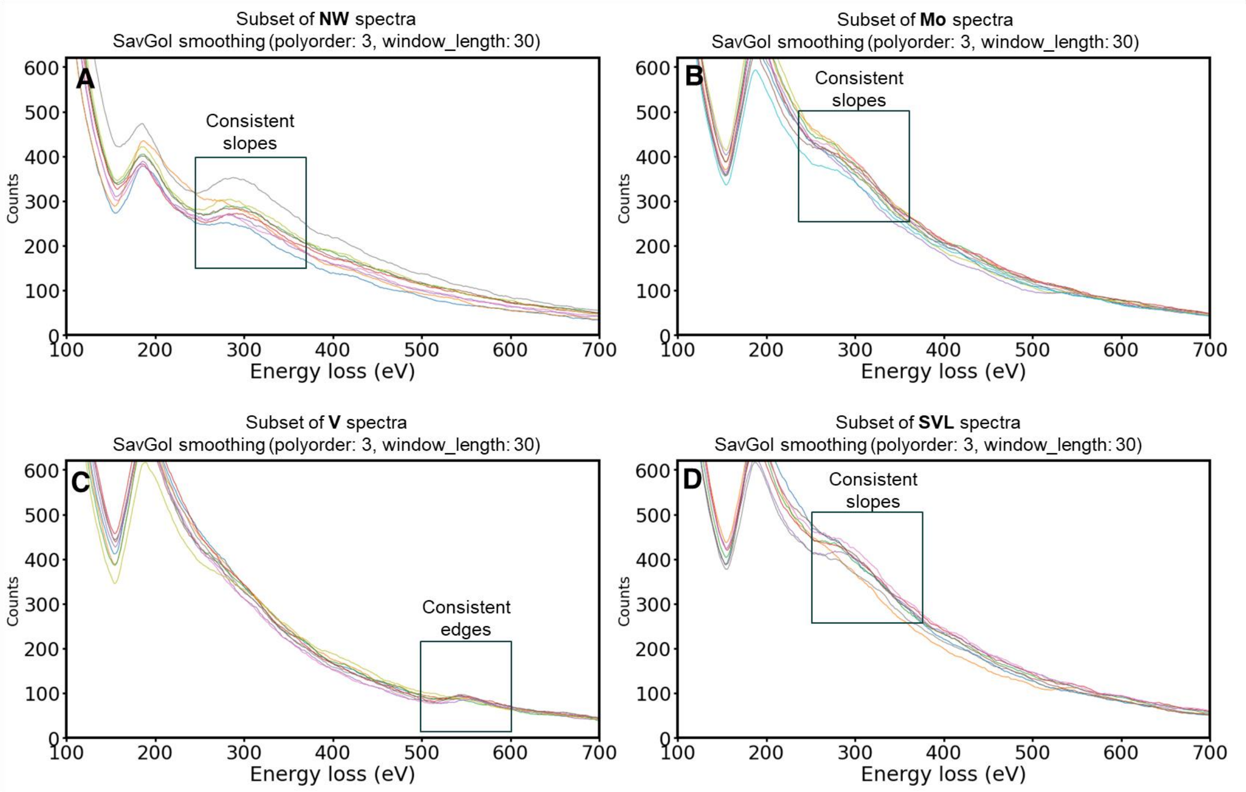

Figure S5. Subset of individual core-loss spectra for different classes showing the consistency of slopes and edges within a given class. All spectra are Savitzky-Golay filtered by the specified parameters, which is reasonable due to the extremely low readout noise on direct electron detection. Boxes indicate highlighted spectral regions where differences in electronic structure are observed amongst different classes. Spectral differences between NW spectra and Mo spectra are clearly seen in comparing **A** and **B**, particularly the slopes in the boxed regions. Core loss vanadium fingerprint is repeatably observed in **C** many times. Core loss spectra of SVLs in **D** are less distinctive relative to Mo.

DATA REPOSITORY

The as-acquired live experimental data may be accessed via:

https://doi.org/10.5281/zenodo.10798656
